# Impact of sleep disordered breathing on postoperative atrial fibrillation in patients who underwent cardiac surgery: a meta-analysis

**DOI:** 10.1080/07853890.2022.2143555

**Published:** 2022-11-09

**Authors:** Zhenni Chen, Rui Zhang, Xueru Hu, Chun Wan, Yongchun Shen, Jiangyue Qin, Lijuan Gao, Jing Zhu

**Affiliations:** a West China School of Medicine/West China Hospital of Sichuan University and West China Tianfu Hospital of Sichuan University, Chengdu, China; b Information Center, West China Hospital of Sichuan University, Chengdu, China; c Department of Respiratory and Critical Care Medicine, West China Hospital of Sichuan University, Chengdu, China

**Keywords:** Sleep disordered breathing, postoperative atrial fibrillation, cardiac surgery, meta-analysis

## Abstract

**Objective:** An increasing number of studies suggest that sleep disordered breathing (SDB) may be associated with postoperative atrial fibrillation (POAF), but these studies present discrepant results. Thus, this meta-analysis aimed to synthesize the data associating SDB with POAF in patients who underwent cardiac surgery.

**Methods:** A literature search was performed in the Scopus, PubMed, Web of Science, EMBASE, CENTRAL, Weipu, Wanfang Data, and China National Knowledge Infrastructure databases before August 2022. Data were extracted, and the strength of the relationship between SDB and the risk of POAF was evaluated using odds ratio (OR) and 95% confidence intervals (CIs). All statistical analysis was carried out using the Stata 12.0 software.

**Results:** A total of 24 studies with 660,685 subjects were included in current meta-analysis. SDB was significantly associated with the risk of POAF in the patients who underwent cardiac surgery (OR = 1.49; 95% CI, 1.30–1.70; *p* < .001). Next subgroup analysis revealed that such association may be increased in the group with medical equipment-measured SDB (OR = 2.27; 95% CI, 1.59–3.23; *p* < .001), prospective studies (OR = 2.17; 95% CI, 1.55–3.03; *p* < .001), patients without a previous history of atrial fibrillation (OR = 2.04; 95% CI, 1.47–2.82; *p* < .001), and patients who received a coronary artery bypass graft (OR = 2.10; 95% CI, 1.45–3.05; *p* < .001). No publication bias was identified.

**Conclusion:** The results of meta-analysis support that SDB may be associated with an increased risk of POAF in patients who had undergone cardiac surgery, and these results should be confirmed in more rigorously designed studies.

**KEY MESSAGES**
- Patients with SDB who underwent cardiac surgery showed increased risk of POAF.
- The relationship between SDB and POAF should be explained with caution with the consideration of various covariate.
- The effect of pre-treatment of SDB on POAF should be examined in future.

## 1. Introduction

Postoperative atrial fibrillation (POAF) is a usual clinical complication of cardiac surgery, which indicates unfavorable clinical outcomes and places a heavy burden on patients [1–3]. Pooled data from various studies suggest that the incidence of POAF is approximately 35% in cardiac surgery cases, peaks on postoperative day 2, and depends on arrhythmia definition, cardiac surgery type, and arrhythmia surveillance method [1–3]. According to the report of Kosmidou et al. the presence of POAF was associated with longer hospitalization duration, a fourfold increase risk of stroke in 3 years, and threefold increase risk of all-cause mortality during all the follow-up period [4]. Schwann et al. also reported that POAF is associated with unfavorable clinical outcomes principally caused by increased intermediate-term cardiovascular and cerebrovascular mortality even after 15 years’ follow-up [5]. In addition, POAF contributed to the increased medical resource utilization and medical costs for patients who underwent cardiac surgery, and the onset of POAF led to an extral medical cost of $10,000 to $11,500 for each patient [6]. Thus, the development of POAF places a heavy burden on both patients and societies, and clinicians are still searching for a reliable solution to prevent POAF. However, the pathogenesis and risk factors of POAF are extremely complicated and have not been fully explained.

An increasing number of studies observed a potential relationship between sleep disordered breathing (SDB) and POAF, especially for obstructive sleep apnea (OSA). A previous study reported that SDB is common among patients who underwent cardiac surgery (26/89, 29%) and might be strongly associated with arrhythmia in such group of patients [7]. SDB, mainly OSA, is featured by sporadic hypoxia, hypercarbia, activation of sympathetic nerve, and release of catecholamine resulted from arousal episodes, which may contribute to POAF [8]. OSA may contribute to POAF through both structural and electrical effects, including negative intrathoracic pressures, left ventricular dysfunction, increased proinflammatory status, and variations in autonomic tone [9–10]. Based on these findings, an increasing number of clinical studies have been performed to assess the relationship between whether SDB and POAF in subjects who have undergone cardiac surgery and whether SDB is a risk factor for POAF, but with contradictory results [11–13]. For better understanding of the relationship between SDB and POAF in the cardiac surgery population, current study used a standard method of meta-analysis to summarize the overall association between SDB and POAF based on the current available publications, following the guidelines in the Meta-Analysis of Observational Studies in Epidemiology statements for reporting systematic reviews and meta-analyses and Preferred Reporting Items for Systematic Reviews and Meta-Analyses [14].

## 2. Methods

### 2.1. Strategy of literature search

Literature search was performed in the Scopus, PubMed, Web of Science, EMBASE, Cochrane Central Registry for Controlled Trials (CENTRAL), Wanfang Data (http://www.wanfangdata.com.cn/index.html), Weipu databases (http://www.cqvip.com/), and China National Knowledge Infrastructure (https://www.cnki.net/) for related articles published before August 2022 without language restriction. Take PubMed for example, the search strategy used a combination of Medical Subject Heading (MeSH) terms and/or text words as followed: (Sleep Apnea Syndromes (MeSH) OR Sleep Apnea, Obstructive (MeSH) OR Sleep-disordered Breathing OR Sleep Apnea OR Obstructive Sleep Apnea OR Sleep Apnea Hypopnea Syndrome OR Obstructive Sleep Apnea Hypopnea Syndrome OR OSA OR OSAHS) AND (Coronary Artery Bypass (MeSH) OR Cardiac Surgical Procedures (MeSH) OR Coronary Artery Bypass Graft OR CABG OR Coronary Artery Bypass Grafting OR Cardiac Surgery OR Cardiovascular Surgery OR Coronary Artery Bypass Surgery) AND (Arrhythmias, Cardiaciac (MeSH) OR Atrial Fibrillation (MeSH) OR Postoperative Atrial Fibrillation). The search strategy in Chinese was presented in Supplementary Material 1. The references of included studies and review articles were checked manually to find additional studies.

### 2.2. Selection of studies

Studies that (1) presented data on the relationship between SDB and POAF on patients underwent cardiac surgery; (2) evaluated SDB status before elective cardiac surgery; and (3) recorded POAF using during the postoperative hospital stay or follow-up were included the meta-analysis. SDB was defined by the medical equipment examination, questionnaire, or review of medical record; POAF was set by continuous electrocardiographic monitoring or review of medical record. Studies that had limited data or review/abstract/letter articles were removed. When publications involved the same or overlapping data sets or from the same research group, only the study with the largest number of participants was included. Two independent reviewers (ZC and RZ) selected the eligible studies, and all disagreements were resolved by consensus (ZC, RZ and XH).

### 2.3. Bias risk assessment

Two independent authors (ZC and RZ) assessed the bias risk of the included studies using Newcastle-Ottawa Scale (NOS) [15]. The NOS scale consists of 8 items including three dimensions in cohort studies, selection (ascertainment of exposure, selection of the nonexposed cohort, representativeness of the exposed cohort, and demonstration that the outcomes of interest were not present at the beginning of the study), comparability (with or without control for one confounder), and outcome (outcome assessment, sufficient long follow-up for outcomes, and adequacy of follow-up cohorts). The highest possible NOS score was 9, and a score of ≥6 was considered as with high-quality. The disagreements were solved by consensus.

### 2.4. Data extraction

The clinical and related information of each included article was extracted, containing the names of authors, year of publication, country, number of subjects, diagnostic method for SDB, SDB type, sex, body mass index, study design (prospective/retrospective, cohort/case-control/cross sectional), previous history of atrial fibrillation, cardiac surgery type, POAF measurement, and time of follow-up.

### 2.5. Statistical analysis

The overall association between SDB and POAF was assessed by odds ratios (ORs) and 95% confidence intervals (CIs). Heterogeneity across studies was calculated using the *I*^2^ index. When *I*^2^ >50%, a random effects model was chosen to pool the data, if *I*^2^ <50%, the fixed-effects model was used. Begg’s funnel plot and Egger’s test were performed to evaluate the risk of publication bias, since Egger’s test was reported to be sensitive to examine publication bias [16–17]. Sensitivity analysis was conducted by sequentially removing individual studies and re-calculating the ORs. Stata 12.0 (Stata Corp., College Station, TX, USA) was used to perform all the statistical analysis.

## 3. Results

### 3.1. Clinical characteristics of the included studies

**Figure 1.**
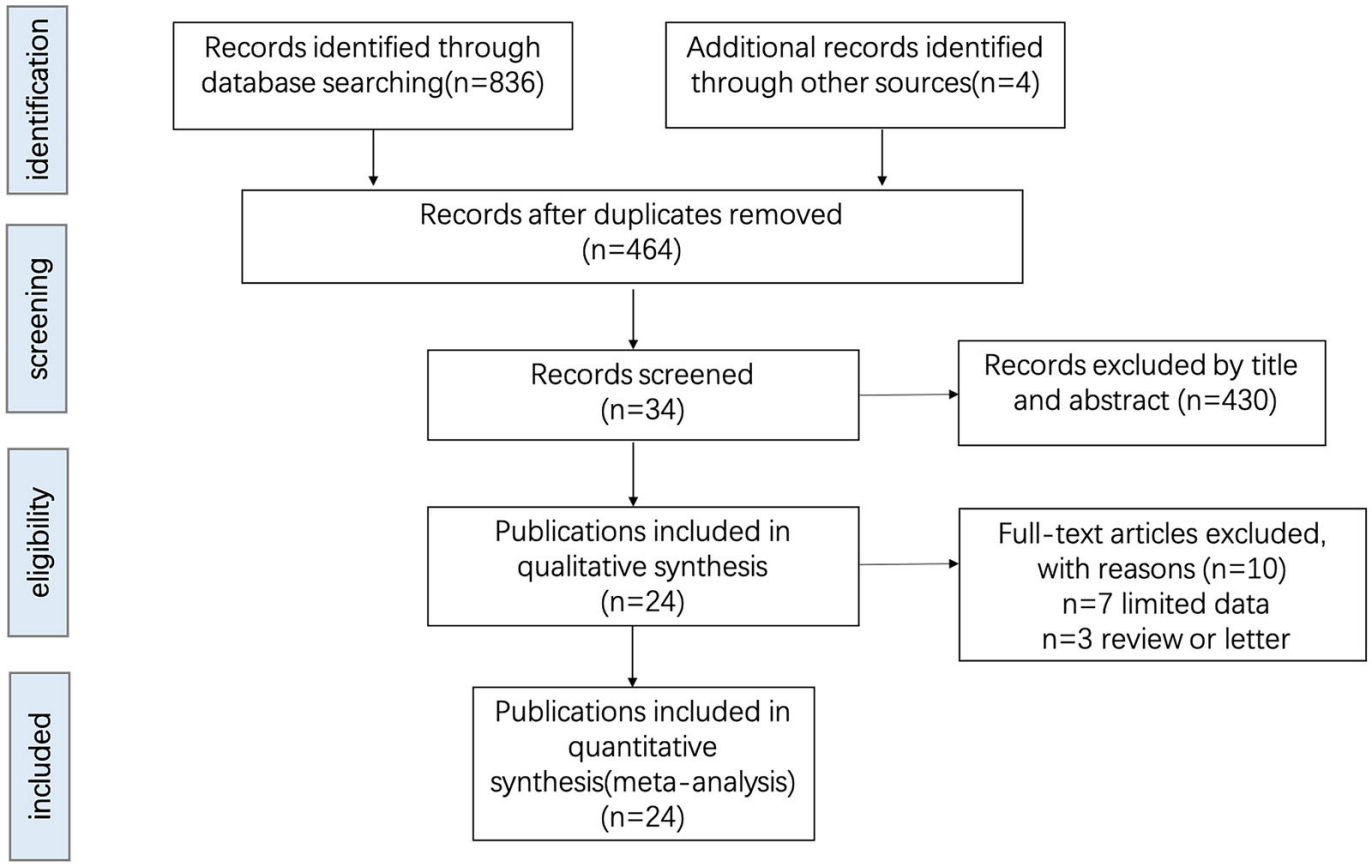
Flow diagram of the study selection.

A total of 24 studies were enrolled for current meta-analysis [18–41]. The selection and inclusion process of the publications is outlined in Figure 1. For this study, 45,361 subjects with SDB and 615,324 subjects without SDB were included. The 24 studies were published between 1996 and 2022, and published across 11 countries, 8 in the United States, 5 in the China, 2 in Sweden and Iran, and 1 each in Brazil, Canada, France, Japan, Turkey, Singapore, and Italy. The clinical summary of the enrolled studies is summarized in Table 1. The bias risk of these studies was evaluated using the NOS, which were all ≥7 (Table 1), suggesting high quality of the included studies and reliable of the meta-analysis results.

**Table 1.** Clinical summary of included studies.

Author (Ref)	Year	Country	SDB measurement	SDB type	Number of Subjects		Age (Year)		Male percentage (%)		BMI (kg/m^2^)		Prospective or retrospective	Study design	NOS score
					SDB	non-SDB	SDB	non-SDB	SDB	non-SDB	SDB	non-SDB			
Mooe et al. [18]	1996	Sweden	PSG	SA	78	39	62.0 ± 7.7	61.3 ± 8.7	NA	NA	NA	NA	P	Cohort	8
Grilli et al. [19]	2007	Italy	PSG	OSA	31	19	68.1 ± 9.5	67.8 ± 9.0	27 (87)	15 (79)	28.1 ± 2.9	26.0 ± 9.0	P	Cohort	7
Sharma et al. [20]	2012	USA	BQ	OSA	81	40	60.2 ± 9.6	59.8 ± 9.7	58 (69)	26 (31)	NA	NA	P	Cohort	8
Mokhlesi et al. [21]	2013	USA	ICD-9-CM	SA	6006	122032	62.1 ± 9.8	64.8 ± 11.1	4991 (83)	84690 (70)	NA	NA	R	Cohort	7
Mungan et al. [22]	2013	Turkey	BQ	OSA	33	40	NA	NA	NA	NA	NA	NA	P	Case-control	8
Amra et al. [23]	2014	Iran	BQ	OSA	25	36	61.1 ± 11.6	57.3 ± 10.5	18 (72)	30 (83)	29.5 ± 3.9	26.0 ± 3.7	P	Cohort	8
van Oosten et al. [24]	2014	Canada	BQ, PSG	OSA	132	145	63.7 ± 10.7	66.4 ± 9.9	106 (80)	110 (76)	31.0 ± 6.0	26.9 ± 3.6	P	Cohort	8
Guenancia et al. [25]	2015	France	NA	SA	13	87	NA	NA	NA	NA	NA	NA	P	Cohort	7
Uchôa et al. [26]	2015	Brazil	PSG	OSA	37	30	59.0 ± 7.9	55.5 ± 6.7	31 (84)	19 (63)	29.1 ± 4.4	27.6 ± 3.3	P	Cohort	7
Wong et al. [27]	2015	USA	Medical record review	OSA	72	473	67.8 ± 10.2	65.7 ± 13.9	57 (79)	312 (66)	31.4 ± 7.2	27.3 ± 5.1	R	Cohort	9
Zhao et al. [28]	2015	Singapore	Watch-PAT 200	SA	128	32	61.9 ± 8.8	63.5 ± 8.3	111 (87)	27 (81)	26.1 ± 3.9	23.6 ± 3.7	P	Cohort	9
Ni et al. [29]	2017	China	NA	OSA	90	270	54.1 ± 7.8	52.4 ± 8.1	61 (68)	187 (69)	NA	NA	P	Cohort	7
Sezai et al. [30]	2017	Japan	SAS-2100	SA	689	206	NA	64.9 ± 14.4	NA	138 (60)	NA	21.5 ± 3.5	P	Cohort	8
Karimi et al. [31]	2018	USA	STOP-BANG	OSA	474	1119	NA	NA	NA	NA	NA	NA	R	Cohort	8
Patel et al. [32]	2018	USA	Self-defined score and Medical Record review	OSA	70	139	NA	66 ± 12.6	50 (71)	103 (74)	NA	NA	R	Cohort	7
Feng et al. [33]	2019	USA	ICD-9-CM	OSA	32545	474059	64.5 ± 10.5	66.3 ± 12.2	24977 (77)	320590 (68)	NA	NA	R	Cohort	8
Gali et al. [34]	2020	USA	Medical record review	OSA	2612	5913	65.1 ± 11.3	61.8 ± 14.7	2001 (76)	3639 (61)	33.8 ± 6.7	27.9 ± 5.3	R	Cohort	8
Wang et al. [35]	2020	China	PSG	OSA	49	36	52.5 ± 10.8	42.8 ± 10.8	30 (61)	24 (67)	25.7 ± 2.9	24.9 ± 3.3	P	Cohort	8
Guo et al. [36]	2021	China	PSG	OSA	142	36	NA	55.6 ± 13.2	NA	20 (56)	NA	23.4 ± 3.1	P	Cohort	8
Ma et al. [37]	2021	China	PSG	OSA	41	29	NA	43.2 ± 10.8	NA	9 (31)	NA	25.0 ± 3.3	P	Cohort	7
Yu et al. [38]	2021	China	PSG	OSA	56	43	NA	42.0 ± 12.3	32 (57)	28 (65)	NA	25.3 ± 2.4	P	Cohort	8
Javaherforooshzadeh et al. [39]	2022	Iran	STOP-BANG	OSA	273	33	NA	54.5 ± 12.9	NA	8 (24.2)	NA	27.1 ± 3.6	P	Cohort	9
Peker et al. [40]	2022	Sweden	Home sleep apnea test	OSA	129	18	NA	NA	NA	NA	NA	NA	R	Case-control	7
Wolf et al. [41]	2022	USA	PSG or medical record review	OSA	1555	10450	65.4 ± 9.4	66.6 ± 11.5	1245 (80)	7506 (72)	34.8 ± 7.2	29.2 ± 5.7	R	Cohort	7

BMI: Body mass index; BQ: Berlin questionnaire; NA: Not available; NOS: Newcastle-Ottawa scale; OSA: Obstructive sleep apnea; P: Prospective; PSG: Polysomnography; R: Retrospective; SA: Sleep apnea; SDB: Sleep-disordered breathing.

The incidence of POAF ranged from 8% to 67% in the patients with SDB and from 6% to 42% in the patients without SDB. The definition of atrial fibrillation was mostly based on findings from continuous electrocardiographic monitoring and electrocardiographic examination, while eight studies did not supply such information [20–21,23,26,29,31,36,39]. The most common surgery type was CABG, and eleven studies included other surgery types such as valve replacement [21,27,30–31,33–35,37–39,41]. The information on POAF and cardiac surgery is listed in Table 2.

**Table 2.** Clinical characters of post-operative atrial fibrillation of included studies.

Author (Ref)	Postoperative atrial fibrillation (%)		Definition of atrial fibrillation	Remove patients with atrial fibrillation history	Surgery type	Follow up time
	SDB	Non-SBD				
Mooe et al. [18]	25 (32)	7 (18)	CEM, ECG	NA	CABG	Operation to discharge from hospital
Grilli et al. [19]	5 (16)	3 (16)	ECG	NA	CABG	Operation to discharge from hospital
Sharma et al. [20]	13 (11)	10 (8)	NA	No	CABG	30 days post discharge
Mokhlesi et al. [21]	1,459 (24)	29,897 (25)	NA	NA	CVS	Operation to discharge from hospital
Mungan et al. [22]	19 (58)	14 (34)	CME	Yes	CABG	Operation to discharge from hospital
Amra et al. [23]	2 (8)	2 (6)	NA	NA	CABG	Operation to discharge from hospital
van Oosten et al. [24]	60 (46)	43 (30)	CEM, ECG	No	CABG	Operation to discharge from hospital
Guenancia et al. [25]	8 (62)	26 (30)	CEM, ECG	Yes	CABG	7 days after surgery
Uchôa et al. [26]	10 (27)	0 (0)	NA	NA	CABG	30 days after surgery
Wong et al. [27]	48 (67)	178 (38)	Medical record and ECG	No	CABG, AVR, MVR	Operation to discharge from hospital
Zhao et al. [28]	32 (25)	3 (10)	CEM, ECG	Yes	CABG	Operation to discharge from hospital
Ni et al. [29]	35 (39)	34 (13)	NA	Yes	CABG	Operation to discharge from hospital
Sezai et al. [30]	190 (28)	28 (14)	CME	No	CVS	7 days after surgery
Karimi et al. [31]	202 (44)	382 (34)	NA	Yes	CABG, AVR, MVR, T/PVR	NA
Patel et al. [32]	40 (57)	58 (42)	ECG	Yes	CABG	Operation to discharge from hospital
Feng et al. [33]	13,159 (40)	169,916 (36)	ICD-9-CM based medical record	Yes	CABG, Valvular disease	Operation to discharge from hospital
Gali et al. [34]	825 (31)	1,677 (28)	Medical record review	NA	CABG, Valvular disease	Operation to discharge from hospital
Wang et al. [35]	15 (31)	4 (11)	Medical record review	NA	Septal Myectomy	NA
Guo et al. [36]	40 (28)	10 (28)	NA	NA	CABG	NA
Ma et al. [37]	14 (34)	3 (10)	CEM, ECG	Yes	Modified morrow operation	5 days after surgery
Yu et al. [38]	19 (34)	6 (14)	CME	Yes	Septal myectomy	Operation to discharge from hospital
Javaherforooshzadeh et al. [39]	24 (9)	5 (15)	NA	Yes	CABG, Valvular disease	10 days after surgery
Peker et al. [40]	46 (36)	2 (11)	CEM, ECG	Yes	CABG	30 days after surgery
Wolf et al. [41]	482 (31)	3,219 (31)	Medical record review	No	CABG, Valvular disease	Operation to discharge from hospital

AVR: Aortic valve replacement; CABG: Coronary artery bypass graft; CEM: Continuous electrocardiographic monitoring; CVS: Cardiovascular surgery; ECG: Electrocardiogram; MVR: Mitral valve replacement; NA: Not available; PVR: Pulmonary valve replacement; TVR: Tricuspid valve replacement.

### 3.2. Meta-analysis

**Figure 2.**
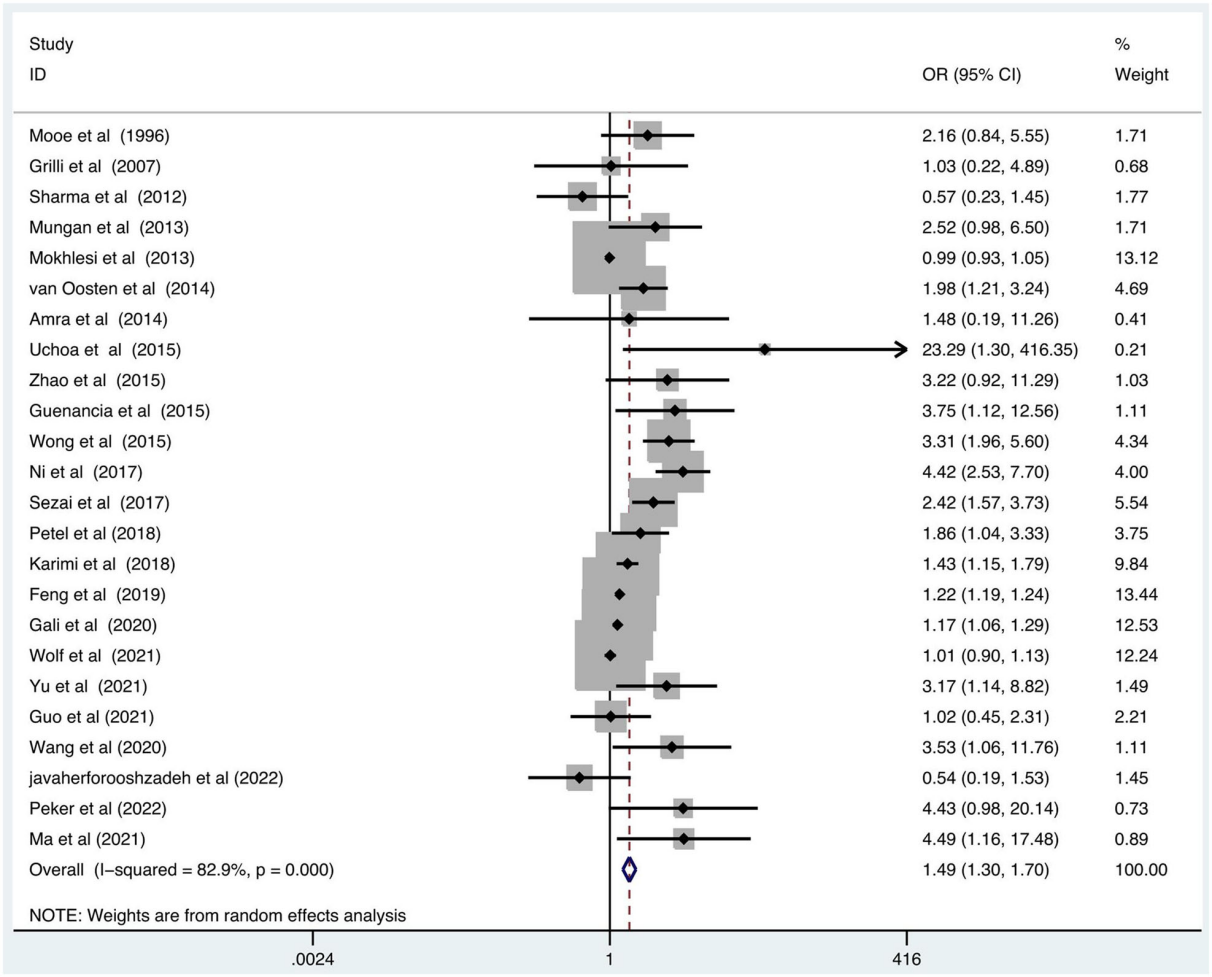
Forest plot showing the pooled POAF odds ratios for SDB. SDB: Sleep disordered breathing; POAF: postoperative atrial fibrillation.

After pooling data from 24 studies, increased risk of POAF was observed in SDB group among the patients who underwent elective cardiac surgery as shown in Figure 2 (OR = 1.49; 95% CI, 1.30–1.70; *p* = .000).

### 3.3. Subgroup analysis

**Figure 3.**
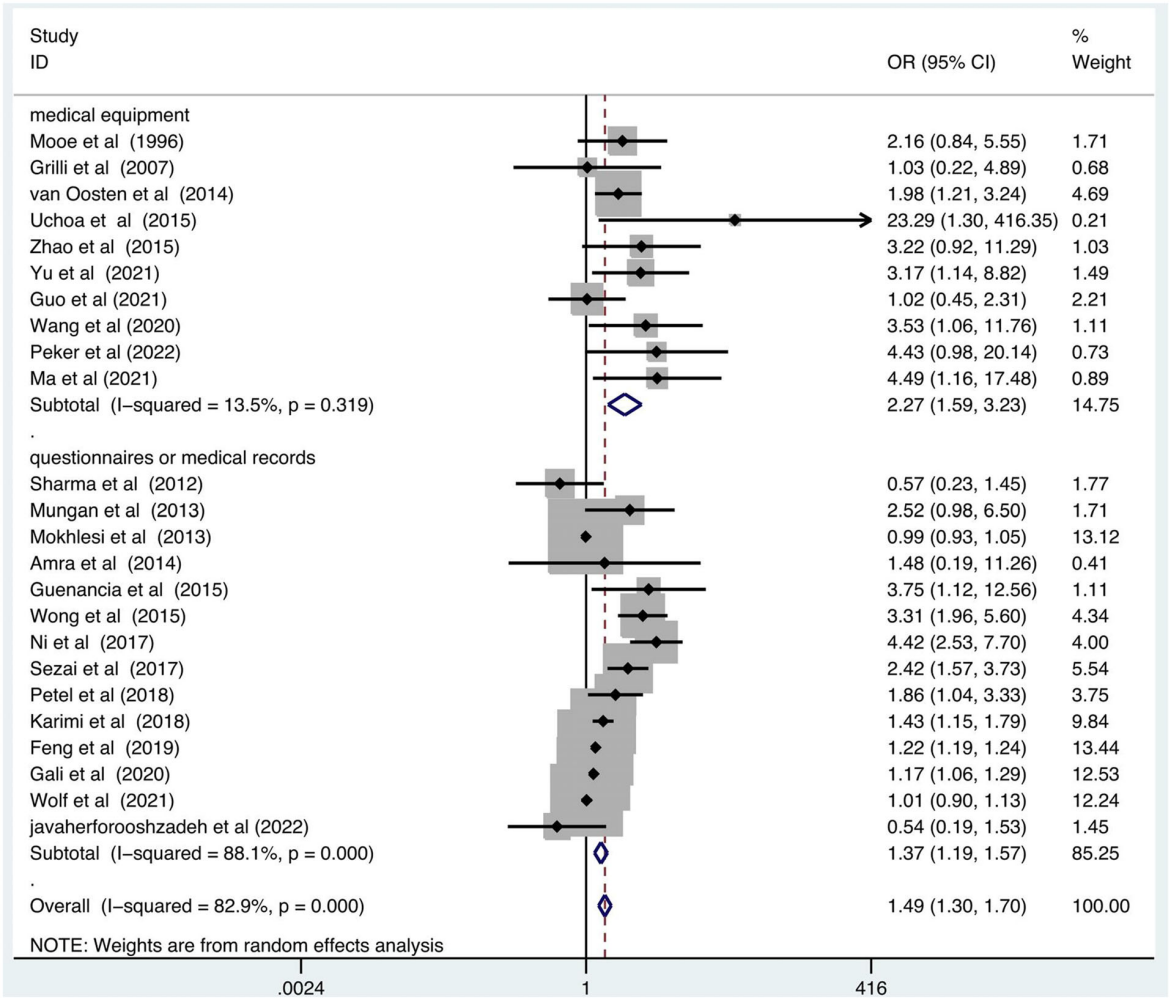
Forest plot showing the pooled POAF odds ratios grouped by type of SDB. SDB: Sleep disordered breathing; POAF: postoperative atrial fibrillation.

**Figure 4.**
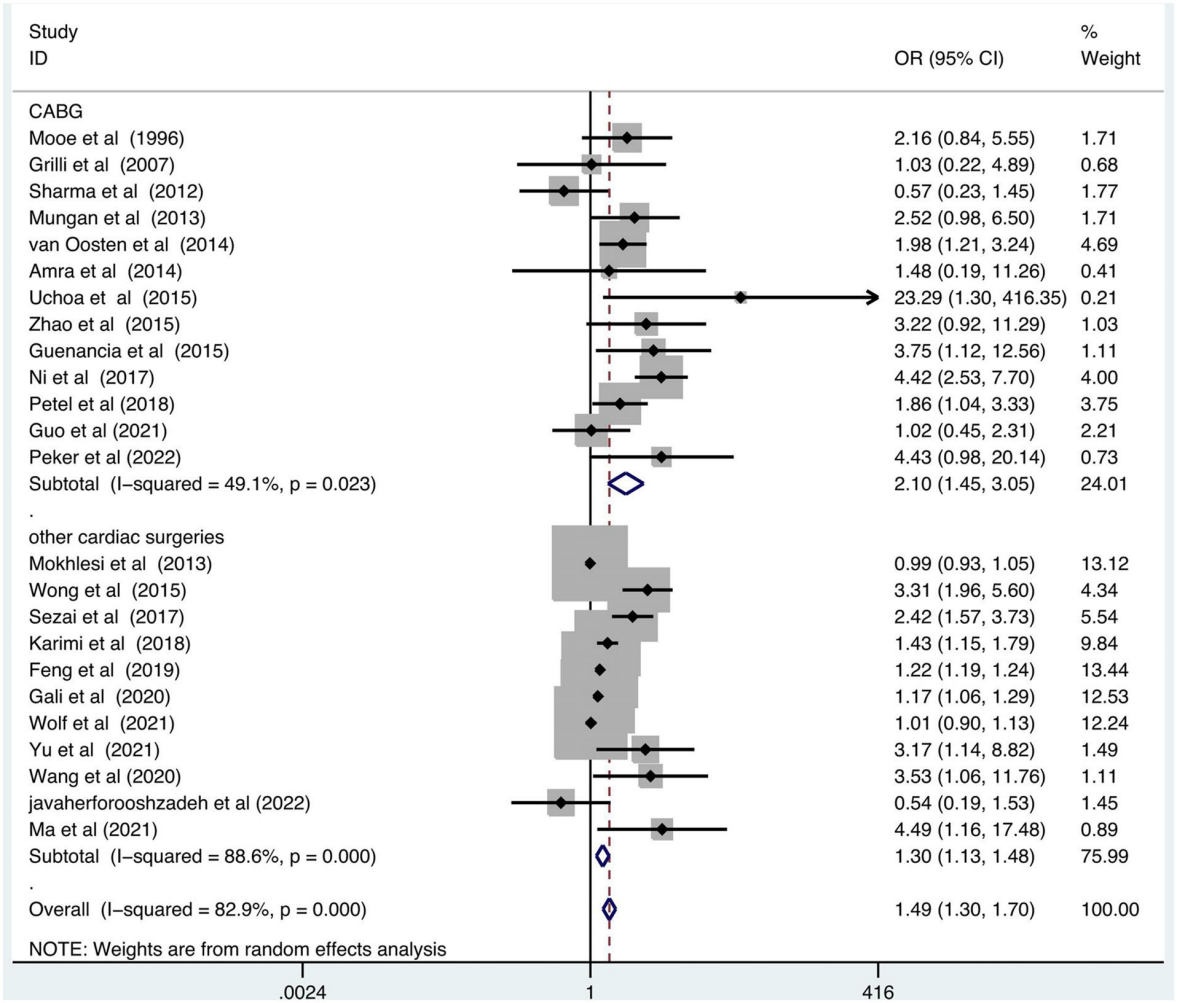
Forest plot showing the POAF odds ratios grouped by surgery type. SDB: Sleep disordered breathing; POAF: postoperative atrial fibrillation.

Next, the subgroup analysis was conducted by SDB measurement method (medical equipment *vs.* questionnaires or medical records), SDB type (OSA *vs.* undefined sleep apnea), study design (Prospective *vs.* retrospective), excluding patients with former history of atrial fibrillation (‘Yes’ *vs.* ‘No’ or ‘Not available’), surgery type (CABG *vs.* mixed cardiac surgeries). The results are summarized in Table 3. The results showed that medical equipment-defined SDB (Figure 3), studies with a prospective study design, excluding a previous history of atrial fibrillation, and CABG surgery type (Figure 4) may increase such association.

**Table 3.** Summary of statistics results of meta-analysis.

Study	No	I^2^	*p* value	Model	OR (95%CI)	*p* value
Overall	24	82.9%	<.001		1.49 (1.30–1.70)	.000
SDB examination						
Medical equipment	10	13.50%	.319	Fixed	2.27 (1.59–3.23)	.000
Questionnaire or medical records	14	88.10%	.000	R	1.37 (1.19–1.57)	.000
SDB type						
Undefined SA	5	84.90%	.000	R	2.03 (1.06–3.89)	.032
OSA	19	76.80%	.000	R	1.51 (1.29–1.76)	.000
Study design						
Retrospective	7	91.05%	.000	R	1.21 (1.07–1.38)	.002
Prospective	17	51.00%	.008	Fixed	2.17 (1.55–3.03)	.000
Remove previous AF history						
Yes	11	77.60%	.000	R	2.04 (1.47–2.82)	.000
No or NA	13	79.90%	.000	R	1.38 (1.14–1.66)	.001
Surgery type						
CABG	13	49.10%	.023	Fixed	2.10 (1.45–3.05)	.000
Other	11	88.60%	.000	R	1.30 (1.13–1.48)	.000

CABG: Coronary artery bypass graft; OR: Odd ratio; OSA: Obstructive sleep apnea; SA: Sleep apnea; SDB: Sleep-disordered breathing.

### 3.4. Sensitivity analysis and publication bias detection

**Figure 5.**
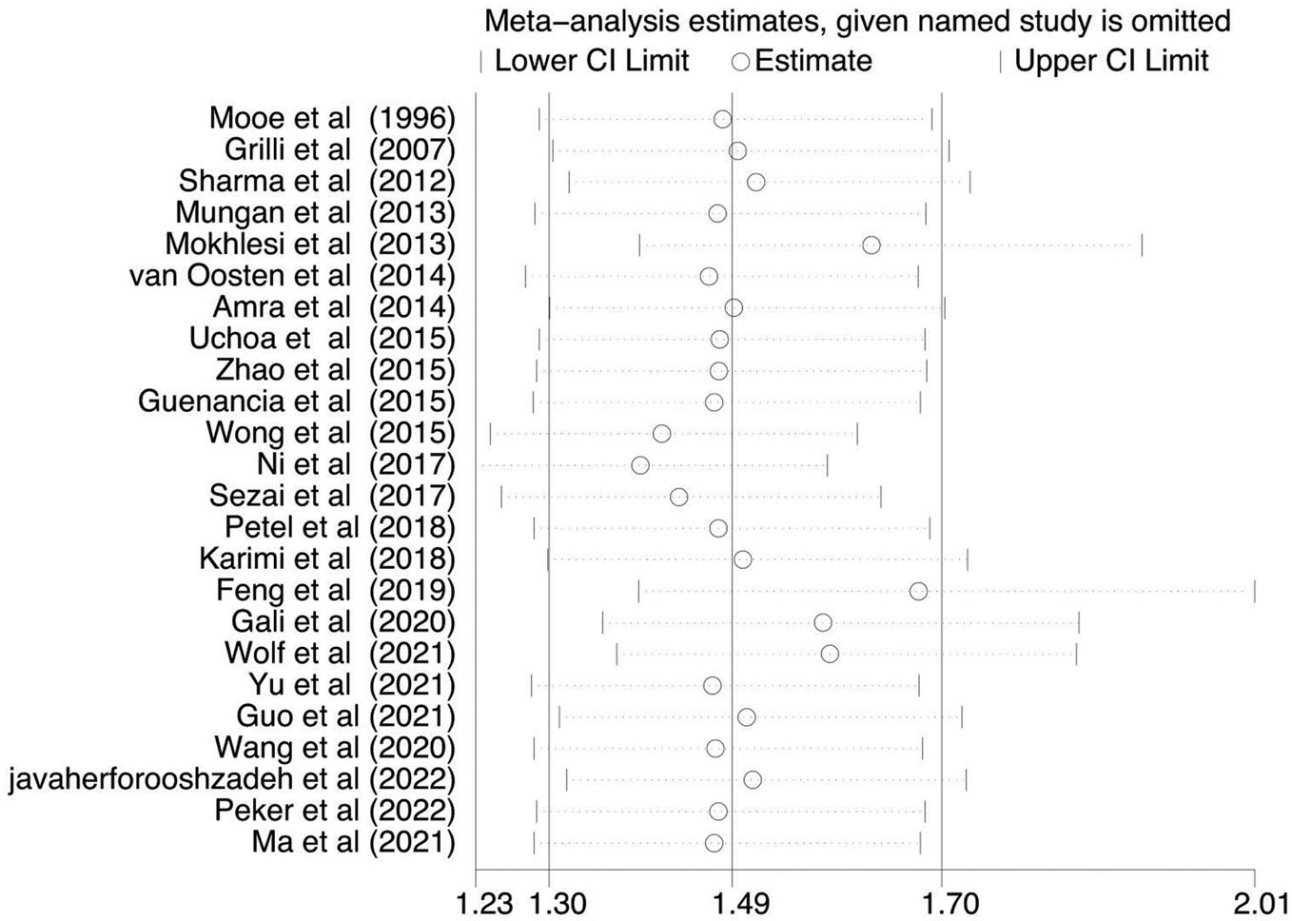
Sensitivity analysis of the included studies for examining the association of SDB and POAF risk. The pooled odds ratio and 95% confidence interval were stability after deletion of each individual study. SDB: Sleep disordered breathing; POAF: postoperative atrial fibrillation.

**Figure 6.**
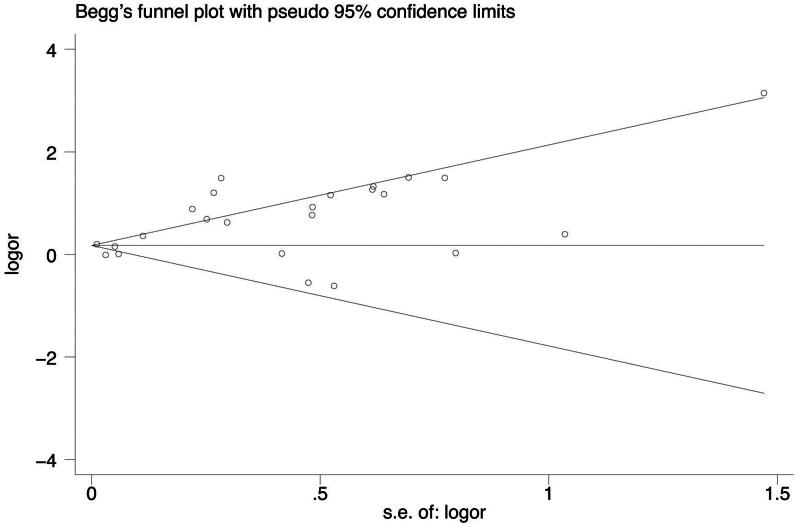
Funnel plot of publication bias.

For sensitivity analysis, we sequentially excluded individual study from the enrolled 24 studies and then recalculated the pooled ORs. Statistically similar results were obtained after sequentially excluding each study, suggesting the stability of the results (Figure 5). Begg’s funnel plot and Egger test result identified a low risk of publication bias (*p* = .039, Figure 6).

## 4. Discussion

An increasing number of studies investigated the potential role of SDB in POAF, but with contradictory results. Identifying patients at high risk of POAF may help improve medical resource utilization, guide preventative treatment, and improve clinical outcomes. In 2014, Qaddoura et al. reported that OSA seems to be an independent risk factor of POAF after elective CABG [42], while this article only included 5 studies, the patients were limited to those who underwent CABG. Currently, more studies have been published regarding the association of SDB and POAF that were not limited to patients who underwent OSA or CABG. In this study, we expanded the scope of SDB and cardiac surgery type, and performed an updated meta-analysis to summarize the overall association between SDB and POAF in 24 studies. Our findings support that SDB may contribute to increased risk of POAF, which should be treated with caution during clinical practice.

In the 24 included studies with a total of 660,685 subjects, we observed that patients with SDB had an increased risk of developing POAF than those without SDB (OR = 1.49; 95% CI, 1.30–1.70; *p* = .000), regardless of the SDB measurement method, previous history of atrial fibrillation, and cardiac surgery type. These results suggested that preoperative intervention for SDB may benefit patients with better clinical outcomes. In a retrospective study with 192 patients from a single-center university hospital, preoperative treatment with positive airway pressure in patients with OSA decreased the incidence rate of POAF after cardiac surgery [43], which suggests that for patients with SDB who will undergo cardiac surgery, preoperative positive airway pressure or other interventions may be needed. In another matched-cohort analysis, preoperative diagnosis of OSA and delivery treatment of continuous positive airway pressure may be useful for reducing the risk of postoperative cardiovascular complications [44]. Thus, systematic screening and timely identification of SDB before cardiac surgery may help to manage such patients comprehensively with preoperative and postoperative interventions to reduce the adverse cardiovascular outcomes.

In the subgroup analysis, first, we observed an increased association in patients with medical equipment-identified SDB (OR = 2.27; 95% CI, 1.59–3.23; *p* < .001). Some investigation used the STOP-Bang score or Berlin questionnaire to identify patients at high risk for OSA rather than the gold standard test, polysomnography, since polysomnography is not available in all levels of hospital and patients that are deemed high risk for OSA rarely have polysomnography testing before cardiac surgery in clinical practice. Although Berlin Questionnaire and STOP-Bang questionnaire may help to risk stratify patients for postoperative complications [45], standard polysomnography test should be applied for future studies to reflect the real status of SDB. Next, we clarified the effect of SDB type on POAF and observed that undefined sleep apnea (may include central sleep apnea and OSA) had increased risk of POAF (OR = 2.03; 95% CI, 1.06–3.89; *p* = .032), and OSA also significantly increased the risk of POAF (OR = 1.51; 95% CI, 1.29–1.76; *p* < .001). Studies showed that central sleep apnea may also be associated with the incidence of atrial fibrillation [46–47], whereas in this meta-analysis, most studies investigated the role of OSA in POAF. In a group of patients underwent cardiac valve replacement surgery, central sleep apnea displayed no significant relation to perioperative events, which can be explained that central sleep apnea is associated with poor heart function, which may play a more important role in occurrence of postoperative complications than central sleep apnea itself [12]. While in another study, central sleep apnea was established as a risk factor for major pulmonary complications after cardiac surgery [48]. Anyway, limited studies focused on central sleep apnea, the incidence of POAF may be associated with the subtype of SDB and should be investigated in further studies.

In addition, for cardiac surgery type, we also identified a more resultful association between SDB and POAF in patients who underwent only CABG surgery (OR = 2.10; 95% CI, 1.45–3.05; *p* < .001), which was also reported by Qaddoura et al. [42]. Another 11 studies included cardiac surgeries other than CABG [21,27,30–31,33–35,37–39,41], including aortic, mitral, and tricuspid/pulmonic valve procedures, and the pooled results showed the significant association between SDB and other mixed cardiac surgeries (OR = 1.30; 95% CI, 1.13–1.48; *p* < .001). Different cardiac surgical procedures may place different influence on the development of POAF, for example, the incidence of POAF increased when CABG was combined with valve replacement surgery [49]. Although the clinical data are limited and the influence of cardiac surgical procedures has not been fully reported and explained, we speculate that the effect of SDB on POAF may be dependent on the cardiac surgery types, and a comprehensive treatment strategy according to differing cardiovascular diseases in the different patient populations should be performed on individual patients.

In sensitivity analysis, no single study showed significant effect on the pooled results. However, we noticed that Feng’s study was a retrospective study with the largest number of research subjects, including 32,545 patients with SDB and 474,059 patients without SDB, which was 77% (506,604/660,685) of all the included subjects. The incidence rates of POAF in the SDB group and non-SBD group were 40% and 36%, respectively, showing no significance without adjustment for confounding factors. Thus, the number of included patients may affect the results, which should be treated with cautious in future studies and more rigorously designed prospective studies should be performed to determine the accurate role of SDB in developing POAF.

During clinical practice, clinicians realize the complications of a disease and various risk factors. A recent systematic review revealed that older age and diseases such as chronic obstructive pulmonary disease and heart failure may all be significant risk factors of POAF in patients who underwent cardiac surgery [50]. The interaction between SDB and other factors should also be considered. Kaw et al. reported that SDB is significantly associated with POAF in their initial analysis, but after adjusting for obesity, such association disappeared [51]. Thus, to determine whether OSA increases the risk of POAF requires correction for all underlying comorbidities and adjustment of potentially confounding factors in the analysis, and patients undergoing cardiac surgery should be evaluated and treated individually.

Our meta-analysis has several limitations that should be explained with caution during clinical practice. First, even after the comprehensive literature search, only 24 publications were enrolled this meta-analysis. The limited number of articles may not be enough to obtain conclusive results. Second, we only included published articles. The omission of unpublished data may cause some bias in the results. Third, we included subjects diagnosed as having SDB based on scores and data from medical records, which may cause patient selection bias. Finally, part of included studies was retrospective studies, thus, further studies with stricter inclusion criteria and study design should be performed. Substantial heterogeneity was identified across studies, and stricter designed studies may help to reduce such bias.

## 5. Conclusion

Taken together, data from this meta-analysis identified a significant association between SDB and the risk of POAF in patients who underwent cardiac surgery. However, more studies should be conducted to validate our results and establish a comprehensive management for patients with SBD.

## Author contributions

Conception and design: ZC, RZ, XH, SY, and JZ; Analysis and interpretation of the data: ZC, RZ, CW, JQ, and LG; Drafting of the paper: ZC, RZ, XH, CW, JQ, and LG; Revising manuscript: YS and JZ; Supervision and administration: YS and JZ; The final approval of the version to be published: all the authors; All authors agree to be accountable for all aspects of the work.

## Supplementary Material

Supplemental Material

## Data Availability

All data used to support the findings of the current study are available from the corresponding authors upon reasonable request.
