# Supplementary material for: Impact of sleep disordered breathing on postoperative atrial fibrillation in patients who underwent cardiac surgery: a meta-analysis
Source: Ann Med. 2022 Nov 9;54(1):3176–87. doi: 10.1080/07853890.2022.2143555 (PMC9661984; doi:10.1080/07853890.2022.2143555)
Supplement: Supplemental Material [file IANN_A_2143555_SM9028.docx]

**CNKI Search strategy:**

(SU= Sleep disordered breathing OR SU= Obstructive sleep apnea hypopneasyndrome OR SU= Obstructive sleep apnea syndrome OR SU= Central Sleep Apnea OR SU= Sleep hypoventilation syndrome OR SU= Sleep related hypoxemia) AND (SU= Postoperative atrial fibrillation OR SU= Postoperative fibrillation) AND (SU= Cardiac surgery OR SU= Postcardiac surgery) AND (SU=Case-control study OR case-control OR Cohort)

**Wanfang Search strategy:**

Subject:( Sleep disordered breathing + Obstructive sleep apnea hypopneasyndrome + Obstructive sleep apnea syndrome + Central Sleep Apnea + Sleep hypoventilation syndrome Sleep related hypoxemia) *Subject:( Postoperative atrial fibrillation + Postoperative fibrillation) *Subject:(Cardiac surgery + Postcardiac surgery) * (case control study OR Case-control OR Cohort)

**Weipu Search strategy:**

M=(Sleep disordered breathing + Obstructive sleep apnea hypopneasyndrome + Obstructive sleep apnea syndrome + Central Sleep Apnea + Sleep hypoventilation syndrome Sleep related hypoxemia)*M=(Postoperative atrial fibrillation + Postoperative fibrillation)*M=(Cardiac surgery + Postcardiac surgery)
